# Computational Fluid Dynamics Analysis of Contrast Agent Distribution in Acute Large Artery Occlusive Stroke: A Preliminary Proof-of-Concept Study

**DOI:** 10.31083/RN54176

**Published:** 2026-10-26

**Authors:** Jiarui Wu, Gelin Xu, Qingyue Gan, Rui Shi, Liming Cao, Pengcheng Fu

**Affiliations:** ^1^School of Medicine, Shenzhen University, 518063 Shenzhen, Guangdong, China; ^2^Department of Neurology, First Affiliated Hospital of Shenzhen University, 518061 Shenzhen, Guangdong, China; ^3^Hunan Provincial Key Laboratory of the Research and Development of Novel Pharmaceutical Preparations, Changsha Medical University, 410219 Changsha, Hunan, China; ^4^Department of Neurology, Shenzhen Second People’s Hospital, 518035 Shenzhen, Guangdong, China

**Keywords:** ischemic stroke, contrast media, hemodynamics, carotid stenosis

## Abstract

**Background:**

Acute ischemic stroke (AIS) resulting from carotid artery occlusion (CAO) represents a significant clinical challenge because conventional imaging modalities often lead to inaccuracies in occlusion length assessment, potentially compromising therapeutic decision-making. We aimed to analyze the distribution patterns of contrast agents using computational fluid dynamics (CFD) simulations in AIS caused by CAO and to mitigate errors in estimating occlusion length.

**Methods:**

Data from four patients with AIS caused by CAO were analyzed. CFD simulations were conducted using postoperative computed tomography angiography (CTA) data to model contrast agent flow, and the results were compared with those of preoperative CTA and intraoperative digital subtraction angiography (DSA).

**Results:**

CTA/DSA demonstrated a flame sign at the ICA origin in Patients 1–3. Patients 1 and 2 had carotid dissection, whereas Patient 3 had a C4-segment embolic occlusion. In Patient 3, DSA showed C1-origin vortices, a horizontal contrast–blood interface, and no distal C1 opacification, features distinct from those in Patients 1 and 2. CFD simulations using the post-recanalization CTA model replicated this contrast distribution, including the vortices and interface; proximal C1 vortex ranges, velocities, pressures, and wall shear stress were comparable, but the mass-weighted average velocity varied over time.

**Conclusions:**

CFD simulations recreated contrast agent distribution during C4 segment occlusion, revealing a high-pressure dead space in the distal C1 segment. This study generated preliminary evidence for understanding contrast agent distribution in CAOs, offering valuable insights for clinical imaging assessments.

## 1. Introduction

Acute ischemic stroke (AIS) accounts for >80% of all stroke cases and is a leading cause of death and disability worldwide [1]. Mechanical thrombectomy is the standard treatment for acute large vessel occlusion (LVO) stroke [2], and procedural success depends on precise imaging of the occluded arteries. Computed tomography angiography (CTA) and digital subtraction angiography (DSA) are the primary imaging modalities for detecting and evaluating LVO. However, discrepancies between the perceived and actual occlusion locations on CTA/DSA complicate treatment selection. Computational fluid dynamics (CFD) uses computer simulations to analyze fluid mechanics [3]. CFD has been widely used to investigate hemodynamics in AIS and LVO [4,5]. CFD is particularly useful for analyzing the contrast agent distribution during LVO imaging. It evaluates contrast agent dispersion in cerebral arteries, enabling quantitative assessment of cerebral hemodynamics. These data support clinical diagnosis and treatment planning.

Contrast agent distribution in AIS caused by large artery occlusion is influenced by occlusion characteristics, collateral circulation, and endovascular intervention [6]. CFD simulations provide quantitative insights into these complex hemodynamic interactions, and may improve imaging interpretation and treatment planning. This study investigated contrast agent flow characteristics and distribution patterns during imaging in AIS caused by carotid artery occlusion (CAO) to improve occlusion length estimation. We hypothesized that contrast agent distribution is primarily governed by hemodynamic principles.

## 2. Methods

### 2.1 Study Patients

Four patients with acute cerebral infarction caused by LVO were consecutively enrolled between April 2023 and March 2024 at the First Affiliated Hospital of Shenzhen University.

### 2.2 Study Design

Data from two patients with dissection-induced proximal internal CAO and two patients with distal internal CAO were compared in this observational study, analyzing differences in DSA imaging characteristics. For the CFD simulation analysis, hemodynamics under distal internal CAO were modeled and simulated using CTA data. The results were compared with preoperative DSA imaging characteristics and those of the CFD analysis.

### 2.3 Inclusion and Exclusion Criteria

The inclusion criteria were imaging-confirmed near-total or complete internal carotid artery (ICA) occlusion and availability of comprehensive clinical and imaging data, including preoperative and postoperative CTA and DSA. The exclusion criteria were incomplete or unclear imaging data and refusal to participate.

### 2.4 Patient Screening and Enrollment Process

A total of 10 patients with LVO were screened; of these, four met the inclusion criteria (imaging-confirmed near-total or complete ICA occlusion with comprehensive pre- and post-operative CTA and DSA data). The cases included two patients with dissection-induced proximal ICA occlusion and two patients with distal ICA occlusion, representing the spectrum of CAO patterns encountered during the study period. Only one patient-specific geometry was used to construct the three-dimensional vascular model for CFD simulation. Postoperative CTA data (after successful recanalization) were used to reconstruct the native vascular anatomy. Different occlusion scenarios were then computationally simulated by virtually occluding the ICA at different levels on this single geometry. The other three patients contributed clinical imaging observations (CTA/DSA findings) but were not individually modeled for CFD analysis.

### 2.5 Clinical Information

A total of four patients were enrolled, and their key clinical information is presented below:

#### 2.5.1 Patient 1

A 30-year-old man was admitted in January 2024 with a 9-hour history of right-sided limb weakness and speech difficulties. The patient had a 2-year smoking history and no other significant medical history. His blood pressure was 142/88 mmHg. Clinical examination revealed mixed aphasia, a shallow right nasolabial fold, rightward tongue deviation, grade 4/5 muscle strength on the right side, and reduced pain sensation in the right upper limb. The National Institutes of Health Stroke Scale (NIHSS) score was 7, and the modified Rankin Scale (mRS) score was 3.

Head computed tomography perfusion revealed reduced blood flow in the left frontal, parietal, and temporal lobes, basal ganglia, occipital lobe, and cerebellum, with a mismatch ratio of 24.8. CTA revealed left ICA occlusion (Fig. 1A). DSA of the common carotid artery (CCA) revealed a flame-shaped remnant (Fig. 1B), whereas ICA DSA revealed a thin line in the C1 segment (Fig. 1C). Successful vascular recanalization was achieved (Fig. 1D,E).

**Fig. 1.**
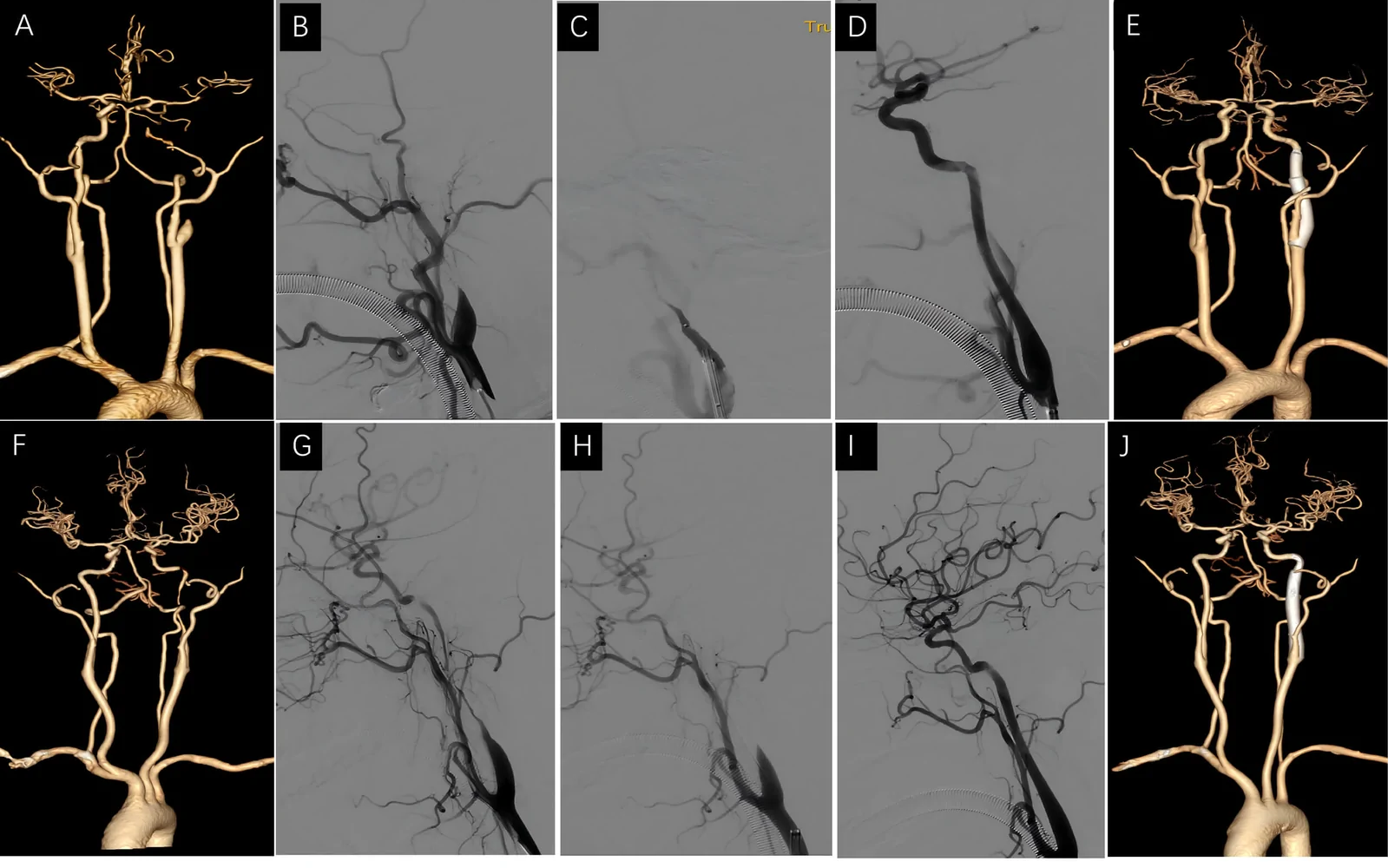
**Pre- and post-procedural CTA and DSA images of patients 1 and 2 undergoing mechanical thrombectomy**. Patient 1 (A–E): (A) CTA showing proximal occlusion of the left ICA. (B) Common carotid angiography revealing a flame-like remnant. (C) ICA angiography demonstrating a slender, elongated appearance in the mid-C1 segment. (D,E) Post-stenting images showing successful recanalization of the vessels. Patient 2 (F–J): (F) CTA showing severe stenosis or possible occlusion of the left ICA C1 segment. (G) CCA angiography showing string-like narrowing of the mid-portion of the ICA C1 segment. (H) After carotid artery stenting, the distal left ICA C1 segment remains occluded. (I,J) Following placement of a second stent in the distal left ICA C1 segment, complete recanalization was achieved. CTA, computed tomography angiography; DSA, digital subtraction angiography; ICA, internal carotid artery; CCA, common carotid artery.

#### 2.5.2 Patient 2

A 44-year-old woman presented with a 2-day history of progressively worsening slurred speech that intensified over the subsequent 18 h, in January 2024. She had no significant medical history, and her blood pressure was 95/50 mmHg. On examination, she exhibited mixed aphasia, grade 1/5 muscle strength in the right upper and lower limbs, and positive right-sided pathological reflexes. Her NIHSS and mRS scores were 14 and 4, respectively. Emergency CTA demonstrated severe stenosis or left ICA C1-segment occlusion (Fig. 1F). DSA confirmed a string-like occlusion in the mid-C1 segment of the left ICA (Fig. 1G). The distal left ICA C1-segment remained occluded after carotid stent deployment (Fig. 1H). A second stent was deployed in the distal left ICA C1 segment, resulting in complete recanalization (Fig. 1I,J). Her NIHSS and mRS scores improved to 0 at discharge.

#### 2.5.3 Patient 3

A 49-year-old man presented in April 2023 with right-sided weakness and aphasia of approximately 6 h duration. The patient had no significant medical history. On admission, the patient was drowsy and exhibited mixed aphasia, a flattened right nasolabial fold, right-sided hemiparesis (grade 2/5 muscle strength), and a positive right Babinski sign. His NIHSS score was 10, and his mRS score was 5. Emergency CTA revealed left ICA occlusion (Fig. 2A). Computed tomography perfusion demonstrated a mismatch ratio of 18.6. DSA of the left CCA revealed a flame sign proximal to the ICA (Fig. 2B), and selective ICA angiography localized the occlusion to the C4 segment (Fig. 2C). Direct thrombus aspiration with intraoperative intravenous tirofiban infusion resulted in left ICA recanalization (Fig. 2D,E).

**Fig. 2.**
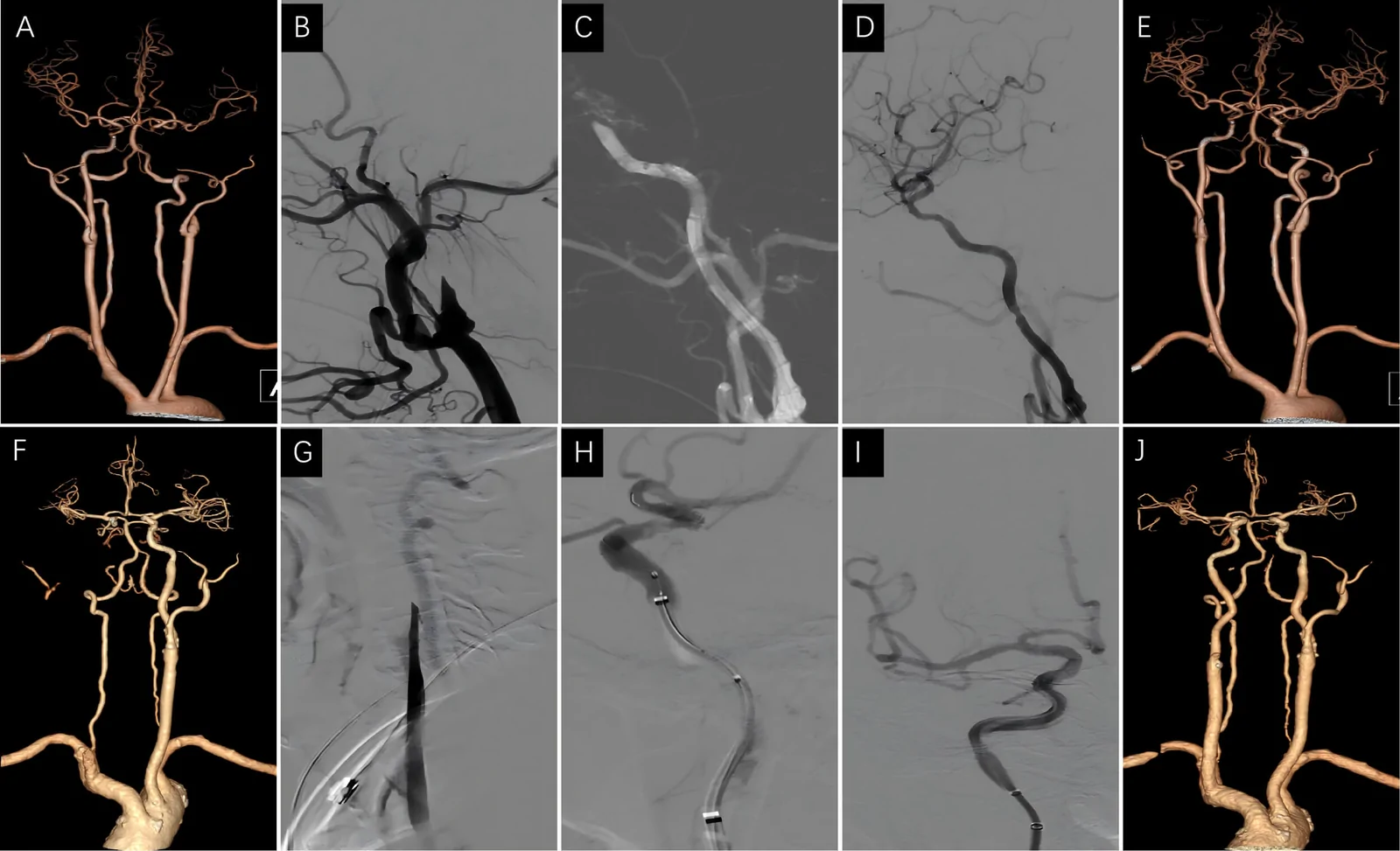
**Pre- and post-procedural CTA and DSA images of patients 3 and 4 undergoing mechanical thrombectomy**. Patient 3 (A–E): (A) CTA showing occlusion of the left ICA. (B) CCA angiography revealing a flame sign at the proximal ICA with a fluid level. (C) Selective ICA angiography demonstrating occlusion in the C4 segment. (D,E) Post-thrombectomy images showing complete recanalization of the vessels. Patient 4 (F–J): (F) Proximal occlusion of the right CCA. (G) CCA angiography showing contrast agent retention in the CCA with a fluid level. (H) Selective ICA angiography indicating occlusion in the C7 segment. (I,J) Post-thrombectomy images showing complete recanalization of the ICA.

#### 2.5.4 Patient 4

An 81-year-old man was hospitalized for cholecystitis in March 2024. The patient suddenly developed left-sided weakness and slurred speech with a 1.5 h history. His medical history included >10 years of hypertension and cardiac stenting performed 1 month earlier. Anticoagulant and antiplatelet therapy was discontinued during hospitalization. The patient was alert but unable to speak spontaneously, and both eyes deviated to the right. The patient had grade 4/5 muscle strength in the right upper limb and painful contractions in the right lower limb. Muscle strength in the left upper and lower limbs was grade 0/5, with positive pathological signs on the left side. The NIHSS score was >11, and the mRS score increased from 0 before symptom onset to >2 after symptom onset. Emergency CTA revealed proximal right CCA occlusion (Fig. 2F). CCA DSA showed contrast agent retention (Fig. 2G), whereas selective ICA DSA demonstrated C7-segment occlusion (Fig. 2H). The patient underwent balloon angioplasty, thrombectomy, and stenting, resulting in successful recanalization of the occluded artery (Fig. 2I,J).

### 2.6 Equipment and Imaging Protocol

CTA and DSA were the primary imaging modalities. Three-dimensional (3D) vascular models were reconstructed using Mimics Medical 21.0 (Materialise NV Inc., Leuven, Belgium) for CFD simulation. ANSYS Fluent 19.2 (ANSYS, Inc., Canonsburg, PA, USA) was used for CFD simulations, and CFD-Post 2023 R2 (ANSYS, Inc., Canonsburg, PA, USA) was used for post-processing. All patients underwent preoperative and postoperative CTA, and intraoperative DSA was performed to evaluate ICA occlusion level and to identify the dissection origin in the C1 segment. Key imaging findings included the flame sign, vortex formation at the C1 segment origin, and a horizontal interface between the contrast agent and blood. The turbulence model, the multiphase model, and the near-wall mesh settings applied in the CFD simulations are described in Section 2.7.3.

### 2.7 CFD Simulation Modeling

#### 2.7.1 Model Preparation

For a patient with C4-segment occlusion, postoperative CTA data were used to construct a 3D vascular model. Raw CTA data stored in Digital Imaging and Communications in Medicine (DICOM) format, were imported into Mimics Medical 21.0 to generate initial three-dimensional models using threshold segmentation, region growing, manual segmentation, and mask editing. These models were subsequently refined and optimized in 3-Matic Medical 13.0 (Materialise NV, Leuven, Flemish Brabant, Belgium) to generate a geometric model suitable for mesh generation. The centerline ends were trimmed to establish inlet and outlet planes perpendicular to the centerline. Additionally, the ICA was truncated at the C7, C4, C2, and C1 segments before the model was converted to a stereolithography (STL) file. For two-phase flow analysis, a cylindrical section (2-mm diameter) was added at the CCA origin to serve as the contrast-agent inlet.

#### 2.7.2 Mesh Generation

The STL file was imported into ANSYS Fluent 19.2, where the Fluent Mesh tool generated a mesh with polyhedral boundary layers and a hexahedral core. Surface and volume meshes were generated with a minimum element size of 0.3 mm, a maximum element size of 0.8 mm, and a growth rate of 1.2 based on vessel diameter. The boundary layer comprised five polyhedral layers. These settings were selected to resolve the viscous sublayer and flow-separation characteristics. The poly-hexcore method was used to generate the volume mesh. Mesh specifications are presented in Table 1.

**Table 1.** **Computational mesh statistics**.

Model	Cells	Faces	Nodes	Cell zones	Face zones
C1	60,306	281,363	173,004	1	6
C2	75,933	354,127	217,812	1	6
C4	85,221	397,928	245,012	1	6
C7	102,799	479,734	294,988	1	6

CFD simulations were performed to model contrast agent distribution patterns during C4-segment occlusion, focusing on vortex formation at the C1-segment origin, the horizontal interface between blood and the contrast agent, the high-pressure dead space at the distal end of the C1 segment and the associated blood-flow dynamics. The boundary conditions are described in Section 2.9. The analysis focused on the high-pressure dead space at the distal end of the C1 segment and the associated blood-flow dynamics.

#### 2.7.3 Turbulence and Multiphase Flow Modeling

For the two-phase flow analysis of blood and contrast agent at the origin of the C1 segment of the carotid artery, the SST k-ω turbulence model was selected owing to the presence of vortex formation and flow separation. The SST k-ω model blends the k-ω formulation in the near-wall region with the k-ε formulation in the free stream via a blending function, providing superior accuracy in predicting adverse pressure gradients and separation phenomena. A mixture multiphase model was adopted to couple the blood and contrast agent phases, which solves a single momentum equation for the mixture while accounting for interphase slip velocity; this approach is particularly suitable for flows with large density and viscosity disparities. Interphase momentum exchange was modeled using the Schiller-Naumann drag law. The automatic wall treatment intrinsic to the SST k-ω model was employed, which seamlessly blends wall-function and low-Reynolds-number formulations based on local mesh resolution. At peak systolic velocity, the area-weighted average wall y+ was below 1, and the local maximum y+ was approximately 1.5, which was confined to small patches near the bifurcation apex and external carotid artery stump. The first-layer cell centroids therefore lay within the viscous sublayer over almost the entire wall, and the automatic wall treatment operated predominantly in the low-Reynolds-number (wall-resolved) regime rather than relying on wall functions.

### 2.8 CFD Simulation and Numerical Settings

***(1) ***Flow separation and vortex formation occur at the origin of the occluded ICA; therefore, all simulations reported here were performed with the SST k-ω turbulence model described in Section 2.7.3. Although the Reynolds number calculated for the unobstructed common carotid artery (Re = 600–700; Eqn. 1) is below the conventional threshold for laminar flow (Re <2300) [7], the flow within the occluded ICA segment is not laminar, and a turbulence model was therefore required. The CCA diameter was 6.58 mm and the external carotid artery outlet diameter was 3.33 mm. 


(1) Re=ρvD/η

where ρ denotes fluid density, D represents vessel diameter, v is the mean flow velocity across the vessel cross-section, and η denotes dynamic viscosity. 

***(***2***) Newtonian model***: In the Newtonian fluid model, blood was treated as an incompressible, isotropic, and viscous fluid [8,9]. Blood density was set at 1060 kg/m^3 ^[9], and dynamic viscosity of 0.0035 Pa·s [10,11]. The contrast agent had a density of 1356 kg/m^3^ and a dynamic viscosity of 0.0118 Pa·s.

***(***3***) Blood control equation***: Blood flow was governed by the Navier–Stokes equation:


(2) ∇⋅u=0

(3) $$\begin{array}{c} \rho \left[\frac{\partial u}{\partial t}+u\cdot \nabla u\right]=-\nabla p+\nabla \cdot \tau \end{array}$$

where u is the blood-flow velocity vector, p is the pressure, ρ is the blood density, and τ is the stress tensor.

### 2.9 Boundary Conditions

Blood was modeled as an isotropic, adiabatic, incompressible, viscous fluid. The CCA origin was designated as the inlet, with velocity and pressure specified at the inlet and outlet boundaries, respectively. All other boundaries were treated as rigid, no-slip vascular walls. Physiological pulsatile velocity data from a healthy individual were applied at the inlet (Fig. 3) [12]. The distal end of the external carotid artery served as the outlet, with the outlet pressure set to 13,300 Pa. A single contrast-agent pulse was modeled at an injection velocity of 1.6 m/s for 1.4 s. The contrast agent was injected as a pure phase (volume fraction = 1) through a dedicated 2-mm-diameter cylindrical inlet at the CCA origin, mimicking catheter-tip injection during DSA, while blood (contrast volume fraction = 0) entered through the remaining cross-sectional area of the CCA inlet according to the pulsatile velocity waveform; no premixing of the two phases was imposed at the boundary.

**Fig. 3.**
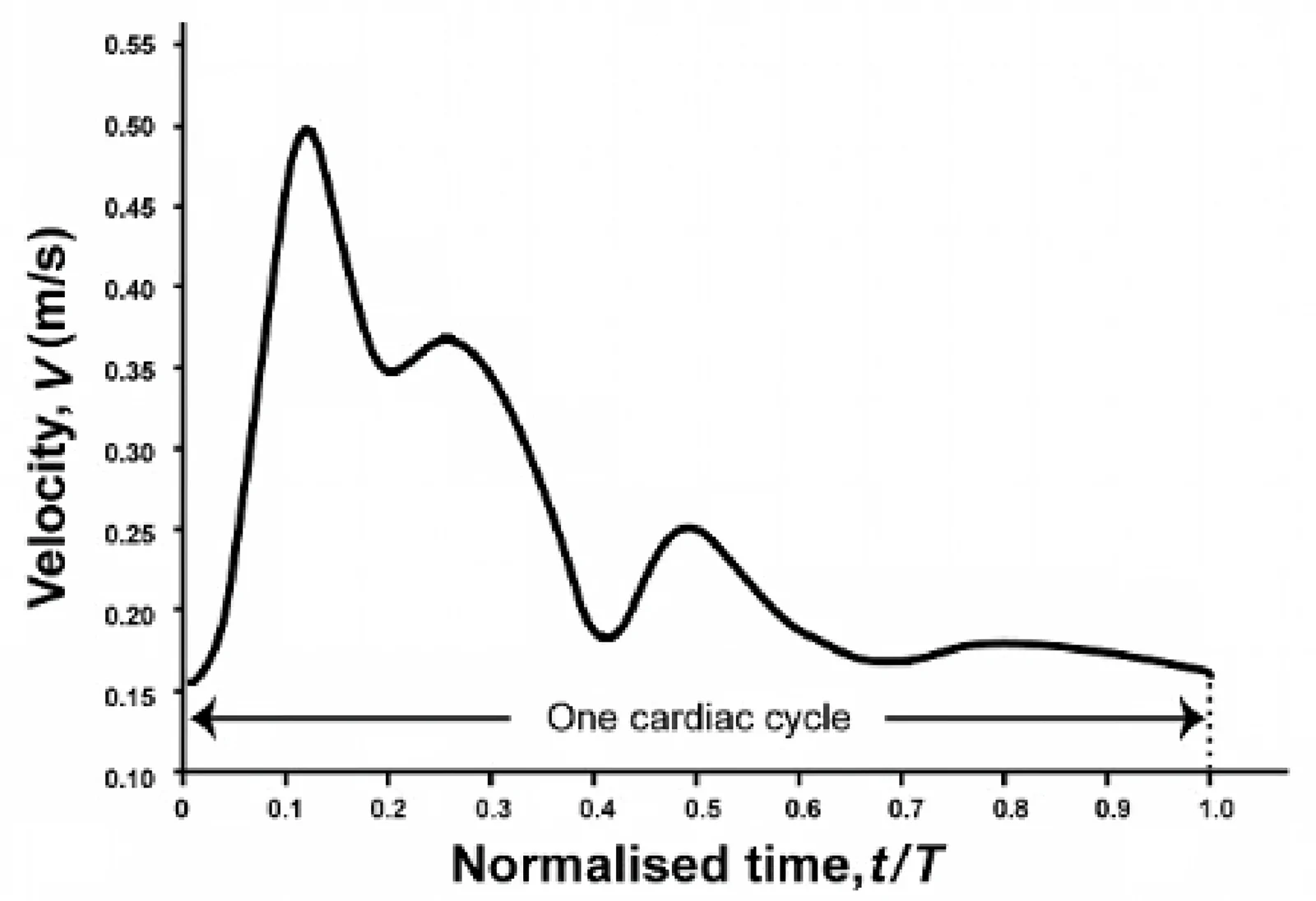
**Velocity waveform for the common carotid artery over one cardiac cycle**.

The vascular wall was modeled as rigid. Numerical data were recorded after three unsteady cycles, beginning with the fourth cycle. Each cardiac cycle lasted 1 s and was divided into 100 time steps. The convergence criterion required the continuity-equation residual to be ≤1 × 10^–4^. In the two-phase flow simulation, the blood inlet and outlet conditions and physical properties were identical to those used previously. The simulation began with a 1-s baseline calculation, followed by a 1.4-s contrast-agent injection. After the injection ceased, the simulation continued for an additional 2 s, resulting in a total runtime of 4.4 s.

### 2.10 Impact of Flow State

To evaluate the effect of flow state on contrast-agent distribution, changes in the liquid interface were recorded at different head positions.

### 2.11 Comparing Occlusion Locations

The ICA was computationally occluded at C7, C4, C2, and C1 segments to simulate different occlusion locations. CFD simulations were performed to analyze contrast-agent distribution under each occlusion scenario. Key parameters, including vortex extent, velocity, pressure, and wall shear stress proximal to the C1 segment, were analyzed and compared. Temporal dynamics were also analyzed by comparing mass-weighted average velocity at different time points to evaluate their effects on contrast-agent behavior.

### 2.12 CFD Post-processing and Flow-Field Analysis

All CFD simulation results were imported into CFD-Post 2023 R2 (ANSYS, Inc., Canonsburg, PA, USA), the post-processing software in the ANSYS 2023 R2 suite, for quantitative flow-field analysis and visualization. Key hemodynamic parameters, including velocity, pressure, wall shear stress, and vortex-core data, were examined at different anatomical locations and time points.

(1) Using a C4-segment occlusion model, single-phase transient vortex-flow analysis was performed on the velocity curve during the fourth cardiac cycle, with selected time points used for further analysis. Velocity profiles, cross-sectional velocity contour plots, and pressure contours were analyzed and compared with preoperative DSA findings at corresponding time points to characterize hemodynamics within the occluded ICA.

(2) Biphasic flow, vortex flow, and transient simulations were analyzed using a C4-segment occlusion model. Following a 14° change in gravitational direction, the volume fraction of the contrast agent was rendered using volume-rendering techniques and compared with preoperative DSA findings obtained before and after head elevation.

(3) Single-phase flow, vortex flow, and transient simulation analyses were also performed using C1-, C2-, C4-, and C7-segment occlusion models. The maximum distribution distance of the contrast agent within the C1 segment, together with distal pressure, wall shear stress, vortex-core dynamics, and vorticity changes at peak vortex flow, was evaluated.

## 3. Results

### 3.1 Clinical Imaging Features (All Patients)

In patients 1-3, CTA and DSA images revealed a prominent flame sign at the ICA C4-segment occlusion site and the dissection origin in the C1 segment. Notably, DSA images of the C4-segment occlusion demonstrated a distinct vortex C1-segment at the origin, with a horizontal interface between the contrast agent and blood. However, no contrast agent was detected in the distal C1 segment. A comparison of CTA and DSA findings in patients with ICA dissection and distal ICA occlusion is presented in Fig. 4.

**Fig. 4.**
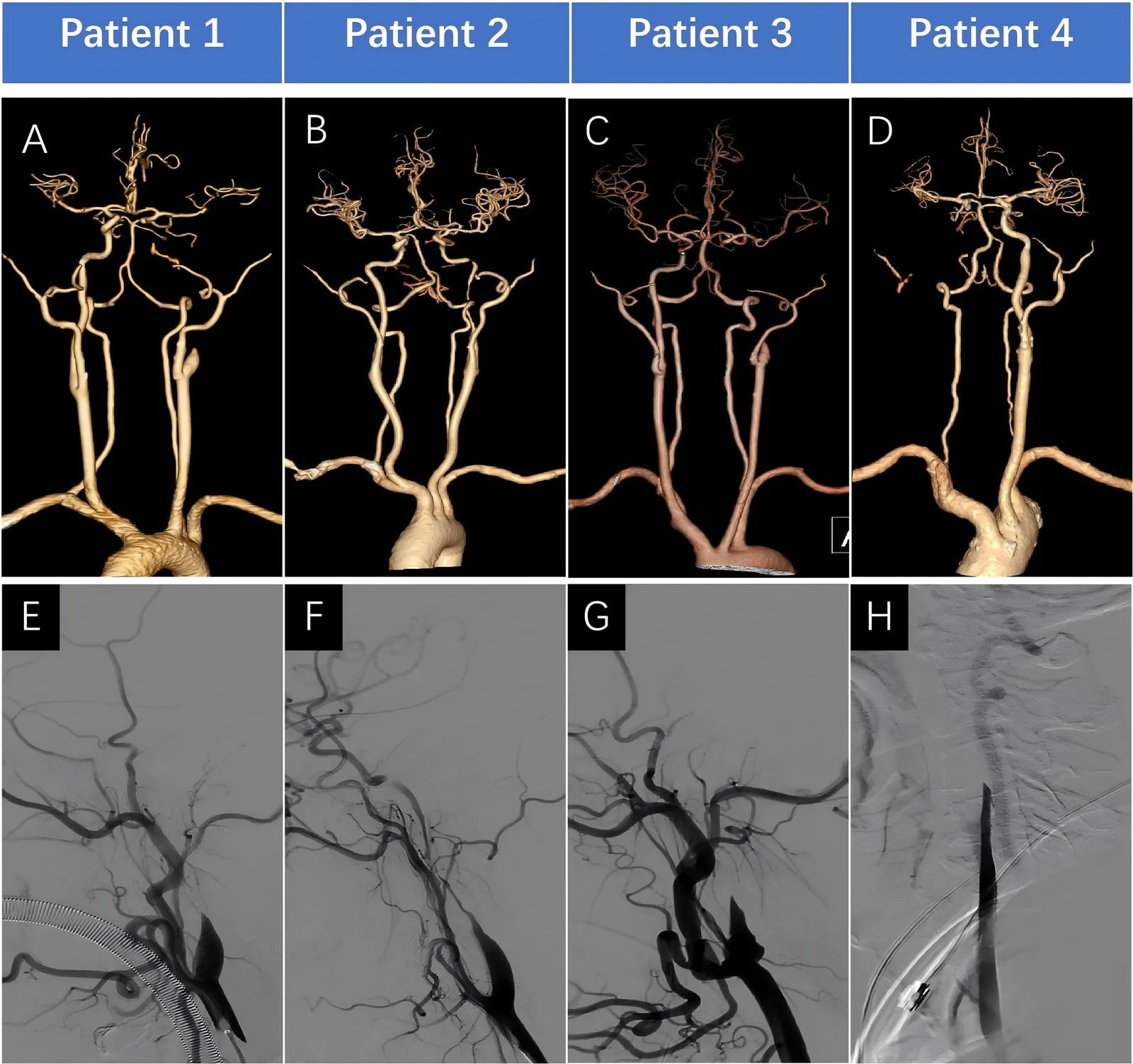
**Comparison of CTA and DSA images in patients with ICA dissection and distal ICA occlusion**. (A–D) show CTA images of patients 1, 2, 3, and 4, respectively. (E–H) show the corresponding DSA images. Patients 1 and 2 had ICA dissection. In patient 2, because there is no complete occlusion, typical linear signs are observed on both the CTA (B) and DSA (F) images. In patient 1, complete occlusion is evident on CTA (A) and common carotid artery DSA (E), which show a residual end resembling a candle flame at the origin of the ICA. However, selective internal carotid angiography reveals linear signs, confirming the diagnosis of dissection. Patients 3 and 4 had distal ICA embolism. In patient 3, the occlusion is located at the C4 segment. CTA (C) and common carotid artery DSA (G) show a residual end resembling a candle flame at the origin of the ICA. However, selective ICA DSA shows that the occlusion does not extend to the C1 segment but gradually approaches the residual end, ultimately confirming its precise location. Patient 4 had distal ICA embolism combined with external carotid artery embolism. Surgical findings confirmed that the occluded segment is at the C7 segment; however, CTA (D) and proximal common carotid artery DSA (H) show a residual end in the common carotid artery.

### 3.2 CFD Simulation Results (Single-Patient Model)

CFD simulation revealed a high-pressure dead space in the distal C1 segment, where no blood flow was observed. The characteristics of vortex flow and distal dead space at the C1 segment on DSA and CFD simulation are presented in Figs. 5-1,5-2.

**Fig. 5.**
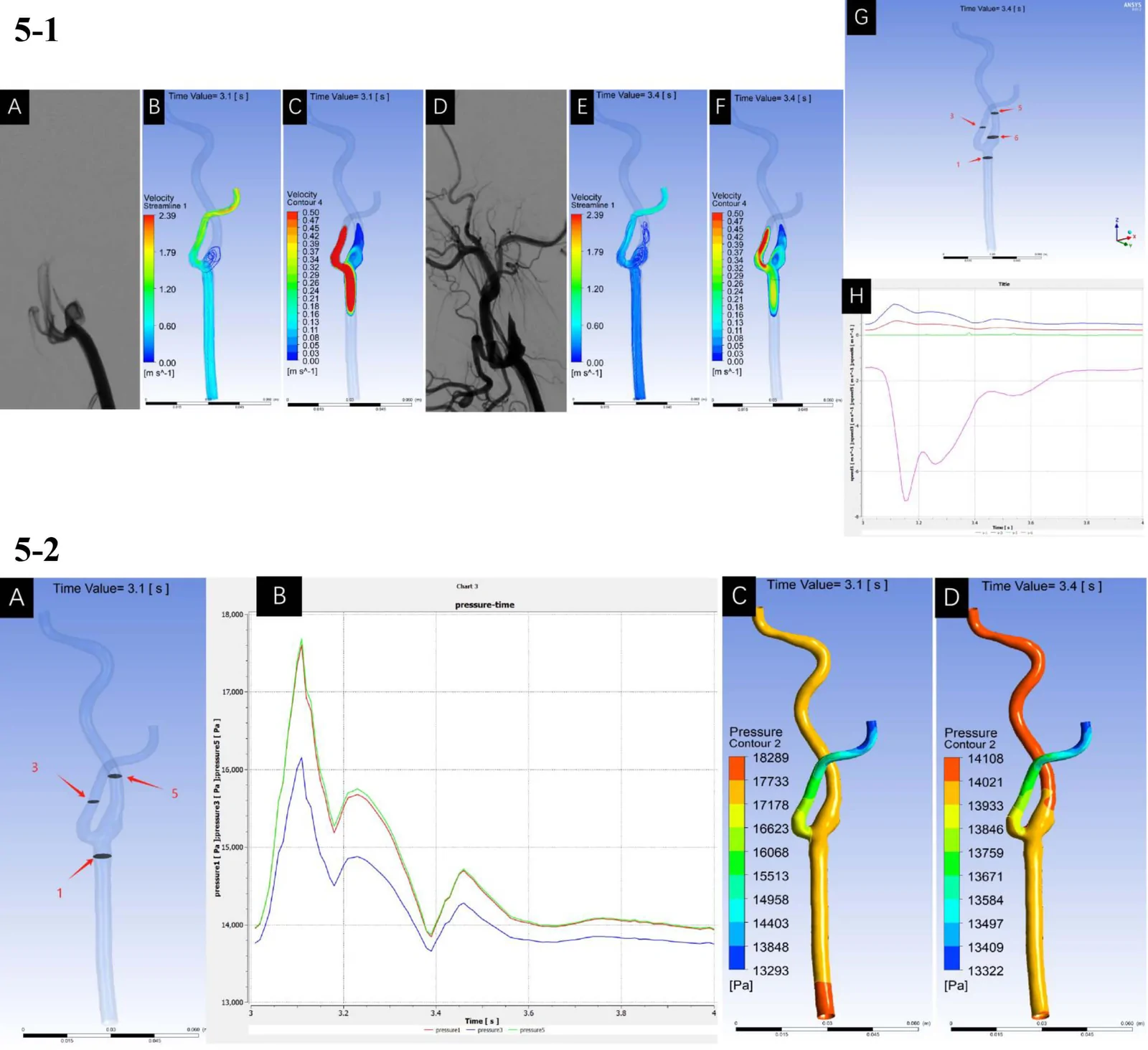
**Characteristics of vortex flow and distal dead space at the C1 segment in DSA and CFD simulations**. Numbered cross-sections established on the vascular model: 1, common carotid artery (CCA); 2, carotid bifurcation (trifurcation plane); 3, internal carotid artery (ICA) at the proximal C1 segment; 4, ICA at the mid-C1 segment; 5, external carotid artery (ECA); 6, ICA at the distal C1 segment. The curves in (H) and (B) are colour-matched to these numbered cross-sections. Fig. 5-1. Changes in CFD-simulated velocity. During the early phase of DSA (frame 13, A), vortex flow is observed at the ICA origin. The CFD velocity profile (B) reveals vortex formation at the early stage of the ICA, with a height comparable to that observed in DSA. Similarly, the velocity vector diagram of the bifurcation plane (C) indicates an elliptical vortex at the ICA origin. In the later phase of DSA (frame 26, D), the contrast agent reaches the farthest point within the ICA, and a distinct blood-contrast-agent interface is observed at the ICA origin. The CFD simulation velocity profile (E) reveals that blood flow reaches its maximum distance in the later stage of the ICA, with a height comparable to that observed in DSA. Similarly, the velocity vector diagram of the bifurcation plane (F) indicates very low blood flow velocity at the distal end of the ICA C1 segment. The temporal variation curve of the mass-weighted average velocity across four cross-sections (G) is shown in (H). Fig. 5-2. Changes in CFD-simulation pressure. Cross-sections were selected from the common carotid artery, ICA, and external carotid artery (A), and the mass-weighted average pressure-time curves for each cross-section are shown in (B). The vessel wall pressure cloud map at the early peak point (3.1 s) (C) indicates elevated pressure throughout the ICA. At the later pressure trough (3.4 s), the vessel wall pressure cloud map (D) again reveals elevated pressure along the entire ICA. CFD, computational fluid dynamics.

#### 3.2.1 Impact of Flow Conditions

Despite head elevation, the liquid interface between blood and the contrast agent remained horizontal, indicating that flow conditions influenced contrast-agent distribution. Contrast-agent distribution in the CFD simulation is presented in Fig. 6.

**Fig. 6.**
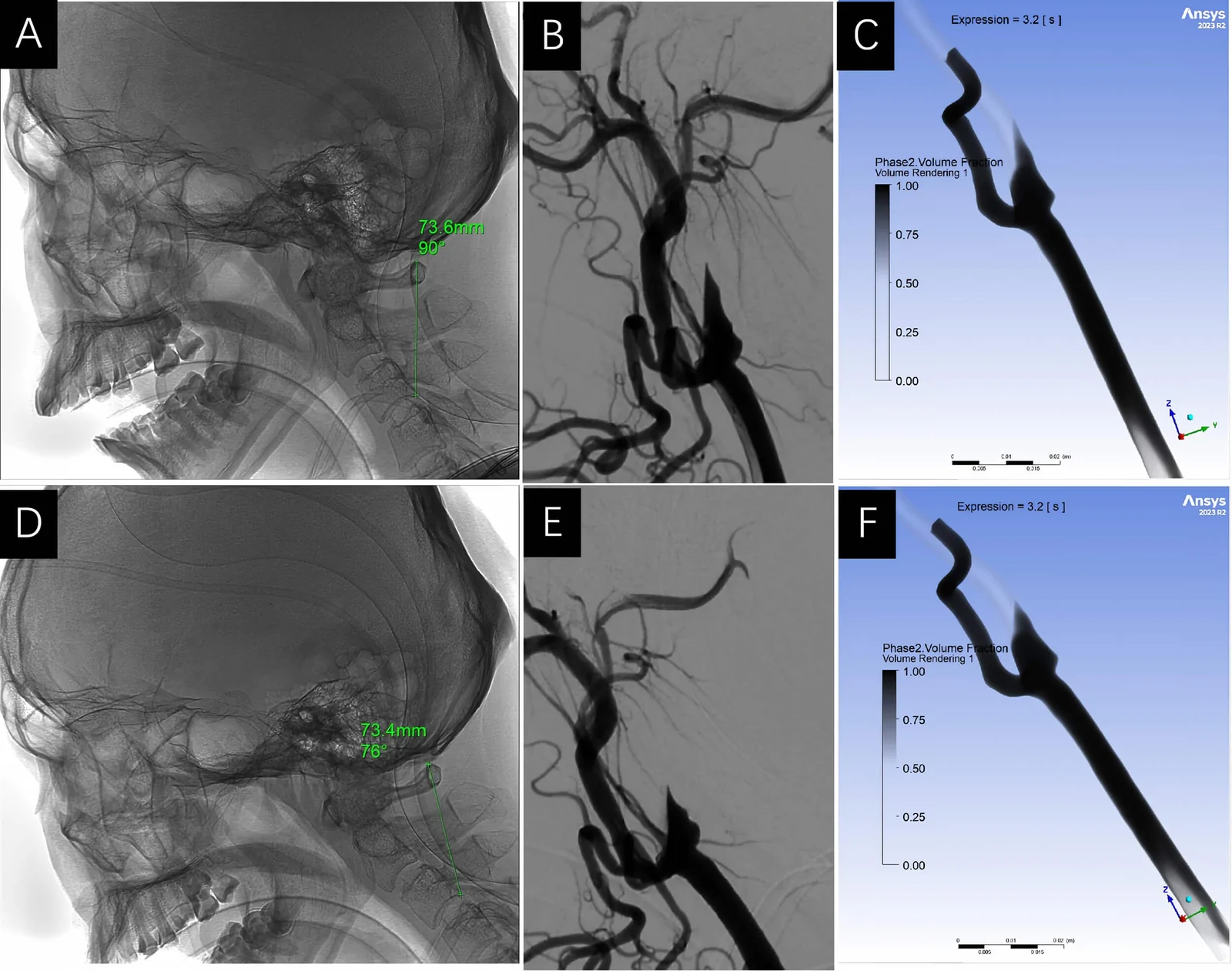
**Contrast agent distribution in CFD simulations**. In the supine position (A), during the later phase of DSA (frame 26, B), the contrast agent reaches the farthest point within the ICA, where it accumulates at the ICA origin, forming a blood-contrast-agent interface. Using a two-phase turbulent flow model for CFD simulations, the volume-rendered image of the contrast-agent volume fraction shows a typical liquid interface at the ICA origin (C), consistent with the DSA findings. When the head is elevated by 14° (D), the blood-contrast agent interface from the later DSA (E) remains visible, and the CFD simulation also demonstrates that the liquid interface changes accordingly with the angle (F).

#### 3.2.2 Comparison of Different Occlusion Locations

Simulations of contrast-agent distribution at different occlusion sites showed that the vortex extent at the proximal C1 segment remained largely consistent across occlusion locations, with stable velocity, pressure, and wall shear stress. However, the mass-weighted average velocity differed markedly across time points, highlighting the importance of temporal dynamics in contrast-agent behavior. CFD simulation results for different occlusion locations are presented in Figs. 7-1,7-2,7-3,7-4,7-5.

**Fig. 7.**
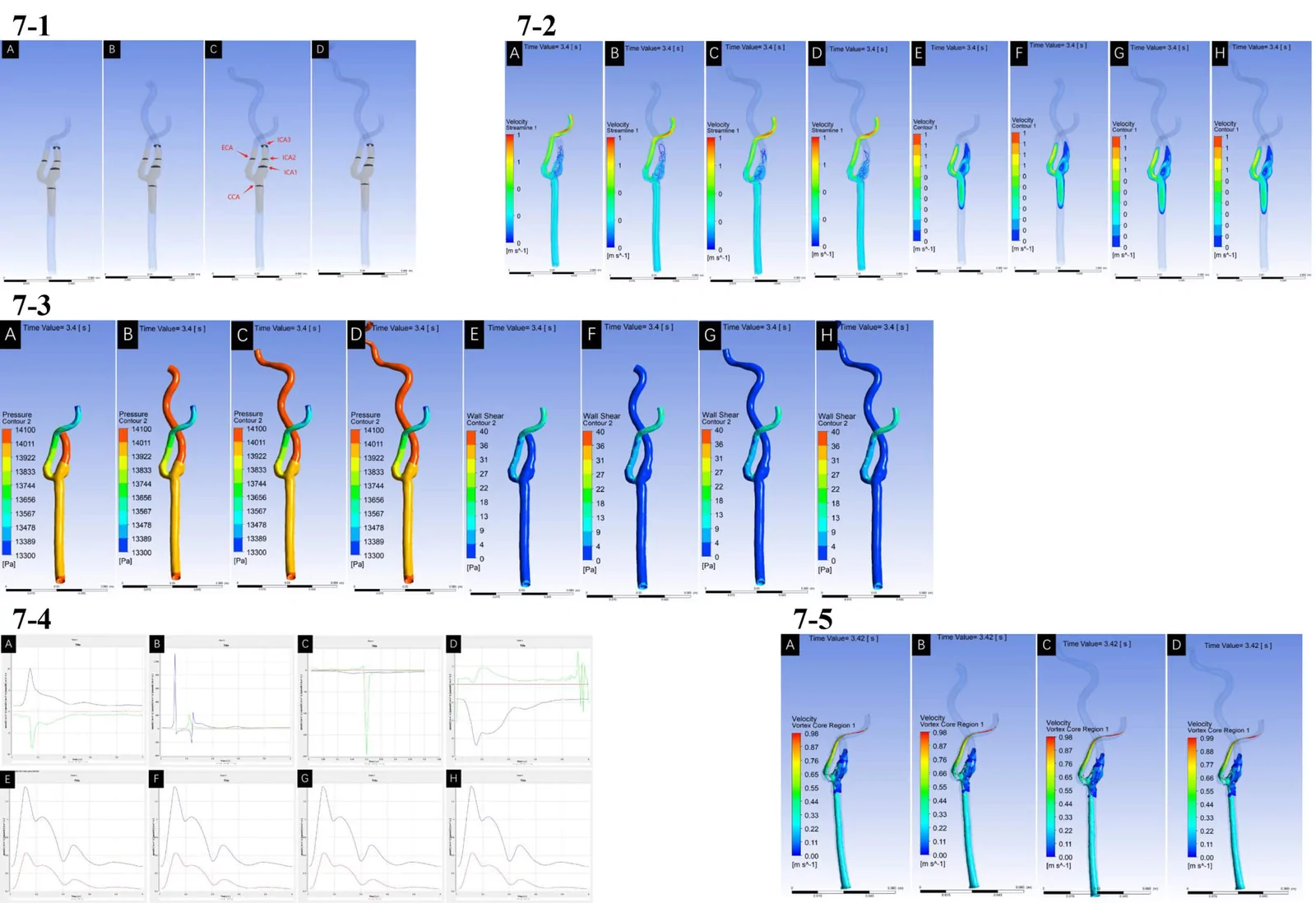
**CFD simulation results at different occlusion locations**. Fig. 7-1. CFD models and cross-sections for different occlusion locations. (A–D) show occlusion model at the C1, C2, C4, and C7 segments of the ICA, respectively. Each model contains cross-sections established at identical anatomical locations. The CCA cross-section is located within the CCA. Three ICA cross-sections (ICA 1, ICA 2, and ICA 3) are positioned sequentially from proximal to distal, beginning at the origin of the C1 segment of the ICA. The cross-section is located within the ECA, and the trifurcation cross-section spans the carotid bifurcation. Fig. 7-2. CFD simulations showing velocity profiles and velocity cloud maps for different occlusion locations. (A–D) present the velocity profiles representing the velocity line graphs and curves of blood flow at the farthest point reached in the ICA following occlusion at C1, C2, C4, and C7, respectively. (E–H) represent the corresponding velocity cloud maps at cross-section 4 when blood flow reaches its farthest point in the ICA after occlusion at C1, C2, C4, and C7, respectively. Fig. 7-3. CFD simulations of pressure cloud maps and wall shear stress cloud maps for different occlusion locations. (A–D) illustrate vessel wall pressure cloud maps, representing the wall pressure distribution when blood flow reaches its farthest point in the ICA following occlusion at C1, C2, C4, and C7, respectively. (E–H) represent the corresponding wall shear stress cloud maps when blood flow reaches its farthest point in the ICA following occlusion at C1, C2, C4, and C7, respectively. Fig. 7-4. CFD simulations of velocity at different cross-sections for different occlusion locations. (A–D) show changes in mass-weighted average velocity over one cardiac cycle at three ICA cross-sections (ICA 1, blue; ICA 2, green; and ICA 3, red) following occlusion at the C1, C2, C4, and C7 segments, respectively. (E–H) show time-resolved mass-weighted average velocity curves over one cardiac cycle at two cross-sections (ECA, blue; and CCA, red) following occlusion at the C1, C2, C4, and C7 segments, respectively. Each point represents the cross-sectional mass-weighted average velocity at that time step; no temporal averaging was applied. Fig. 7-5. CFD simulations of velocity magnitude within the vortex-core region at the peak vortex time for different occlusion locations. (A–D) represent the carotid artery vortex core region at 3.24 s after occlusion at C1, C2, C4, and C7, respectively. Abbreviations: CCA, common carotid artery; CTA, computed tomography angiography; DSA, digital subtraction angiography; ICA, internal carotid artery; ECA, external carotid artery.

## 4. Discussion

This preliminary study investigated contrast-agent flow characteristics in LVO stroke using CFD simulations. The main findings were as follows: First, DSA in patients with C4-segment occlusion demonstrated a vortex at the C1-segment origin and a horizontal interface between the contrast agent and blood. The contrast agent did not reach the distal C1 segment. Second, CFD simulations using the post-recanalization CTA model reproduced contrast-agent distribution during C4 segment occlusion. The simulations reproduced the C1-segment vortex horizontal blood–contrast interface. They also identified a high-pressure dead space in the distal C1 segment. Moreover, head elevation did not alter the horizontal blood–contrast interface. Finally, simulations of different occlusion sites showed a consistent proximal C1 vortex with stable velocity, pressure, and wall shear stress. However, the mass-weighted average velocity varied over time. Overall, these findings improve understanding of contrast-agent distribution in CAOs and may aid clinical imaging assessments.

Reperfusion therapy is an effective treatment for acute LVO stroke and substantially reduces disability. Accurate identification of the occlusion site, residual stump morphology, and underlying cause is essential for selecting the optimal interventional strategy. The key features of intracranial atherosclerotic stenosis stroke, including watershed infarction, truncal-type occlusion [13], and residual stenosis after stenting or three thrombectomy passes [13,14]. Previous studies have described the tapered sign, in which the residual vessel gradually narrows to a pencil-tip appearance. The microcatheter first-pass effect has also been reported [15]. Thrombi associated with intracranial atherosclerotic stenosis may extend both proximal and distal to the occlusion. Our findings highlight the importance of lesion length in predicting treatment outcomes. Lesion length is closely associated with prognosis and reperfusion success [16].

Longer vascular occlusions are associated with greater lesion growth after reperfusion, whereas shorter occlusions generally predict better outcomes [17]. Differentiating intracranial atherosclerotic stenosis-related LVO from cardioembolic stroke relies on accurate identification of the residual stump.

Our findings suggest a CTA/DSA imaging artifact caused by stagnant blood flow within long, branch-free distal occlusions. This produces a contrast-free “dead space”. This dead-space effect may obscure the true occlusion site, impair visualization of the vascular contour, and overestimate occlusion length. Distal ICA occlusion is a representative example of this phenomenon. In such cases, selective catheterization is required to accurately visualize the residual stump.

CFD analysis showed that long, branch-free distal occlusions exhibited high pressure and low wall shear stress. Secondary thrombus formation within the dead space is consistent with clinical observations [18]. CFD simulation demonstrated vortex formation and low-velocity blood flow in the proximal segment of the occluded branch-free ICA. Contrast passage through this region produced a blood–contrast interface, indicating that the contrast-filled region did not represent the true residual stump. Moreover, vortex formation and the blood–contrast interface at the ICA origin remained largely unchanged regardless of distal occlusion length. However, blood-flow velocity varied.

Current models of contrast-agent flow and diffusion can be further improved. Future studies should investigate the physicochemical properties of contrast agents to develop more accurate vascular flow models [19,20]. Blood–contrast agent interactions and temperature-dependent diffusion should also be incorporated to improve simulation accuracy. Finer mesh generation improves representation of vascular geometry and flow characteristics [21,22]. Advances in mesh-generation techniques should further improve CFD accuracy. Patient-specific vascular anatomy and physiology require individualized CFD simulations for accurate assessment of contrast-agent distribution [23]. Patient-specific vascular geometry can be derived from computed tomography (CT) or magnetic resonance imaging (MRI) and incorporated into CFD models. This approach may improve prediction of contrast-agent distribution.

Clinically, the dead space effect has practical implications for endovascular treatment planning. For patients with suspected long-segment ICA occlusion who present with incomplete opacification of distal vascular territory on conventional anterograde DSA, we suggest the following hypothesis-generating considerations that warrant further investigation: First, perform selective ICA angiography with precise catheter placement at the ICA ostium to optimize contrast delivery to the proximal stump and more clearly define the occlusive length based on reflux and collateral filling. Second, obtain delayed-phase angiograms (5–10 s post contrast injection) to visualize slow retrograde collateral perfusion and identify the true distal occlusion boundary that may not be visible on early-phase imaging. Third, combine preprocedural CTA source data with CFD simulations to obtain additional morphological information that may assist in selecting antegrade or retrograde endovascular strategies.

Patients with AIS frequently have comorbidities (e.g., hypertension and diabetes), that can alter vascular morphology, affect contrast-agent distribution, and worsen clinical prognosis [24]. Stroke patients with polyvascular atherothrombotic disease have higher rates of vascular recurrence, stronger associations with inflammatory markers, and poorer short-term prognosis [25]. Recent studies have shown that CTA-based CFD modeling is a reliable, non-invasive alternative to DSA for the morphological evaluation of intracranial arterial stenosis [26].

Using a universal physiological pulsatile velocity waveform derived from a healthy individual as the inlet boundary condition is a notable simplification in this study. The four patients differed considerably in age, baseline cardiovascular status, and comorbidities including hypertension and arteriosclerosis. These factors can substantially alter CCA pulsatile flow. Specifically, arterial stiffening associated with aging and chronic hypertension reduces the pulsatility index, broadens the systolic velocity peak, and increases the resistive index, potentially altering vortex dynamics and contrast-agent distribution. Patient-specific inflow waveforms from phase-contrast MRI or Doppler ultrasound would provide more realistic boundary conditions. However, because of the emergency nature of acute stroke and the retrospective study design, patient-specific CCA hemodynamic data were unavailable. This limitation may have affected CFD accuracy.

### 4.1 Study Limitations

(1) The small sample size (n = 4) precluded statistical analysis and limited generalizability.

(2) Model simplifications included a rigid-wall assumption and the use of a generic, non-patient-specific physiological pulsatile inflow waveform derived from a healthy individual.

(3) The single-center design and specific patient population further limited generalizability.

(4) Differences in patient positioning between CTA and DSA may have introduced simulation discrepancies.

(5) A grid independence study was not performed in this preliminary proof-of-concept study due to computational constraints.

### 4.2 Future Research Directions

Future studies should focus on the following:

(1) Larger prospective studies: Multicenter studies are needed to validate these findings and confirm reproducibility across different occlusion types and locations.

(2) Quantitative CFD-DSA validation: Future studies should compare CFD simulations with DSA using pixel- or region-based analyses to validate model accuracy.

(3) Integration with inflammatory markers: Future studies should investigate correlations between circulating inflammatory biomarkers and CFD-derived hemodynamic parameters.

(4) Longitudinal follow-up: Post-interventional CFD studies with long-term clinical follow-up should evaluate associations between predicted flow patterns, functional outcomes and recurrence.

(5) Methodological improvements: Future CFD studies should incorporate finer mesh densities and rigorous grid-convergence validation. Future studies should incorporate patient-specific inlet conditions.

## 5. Conclusions

CFD simulations successfully reproduced contrast-agent distribution during C4 segment occlusion. These preliminary findings improve understanding of cerebral hemodynamics and may support imaging assessments and treatment planning. As a proof-of-concept study, large validation trials are required. Larger multicenter prospective studies should confirm the reproducibility of contrast-flow patterns. Pixel-level comparison between CFD simulations and DSA images, together with long-term longitudinal follow-up, is needed to strengthen model validation.

## Data Availability

The datasets used and analyzed during this study are available from the corresponding authors on reasonable request.
